# Pan-cancer analysis reveals NAA50 as a cancer prognosis and immune infiltration-related biomarker

**DOI:** 10.3389/fgene.2022.1035337

**Published:** 2022-12-09

**Authors:** Tao Fang, Dingxin Wang, Rongyang Li, Wenhao Yu, Hui Tian

**Affiliations:** Department of Thoracic Surgery, Qilu Hospital of Shandong University, Jinan, Shandong, China

**Keywords:** lung adenocarcinoma (LUAD), N-alpha-acetyltransferase 50 (NAA50), pan-cancer analysis, immune infiltration, biomarker

## Abstract

**Background:** N-Alpha-Acetyltransferase 50 (NAA50) has acetyltransferase activity and is important for chromosome segregation. However, the function and mechanism of NAA50 expression in cancer development was still unclear. Here, we systematically researched the function and mechanism of NAA50 in pan-cancer, and further verified the results of NAA50 in lung adenocarcinoma (LUAD).

**Methods:** In this study, using the online databases TIMER2.0, SangerBox3.0, HPA, UCSC, GEPIA, cBioPortal, UALCAN, TISIDB, CancerSEA and LinkedOmics, we focused on the relevance between NAA50 and oncogenesis, progression, methylation, immune infiltration, function and prognosis. In addition, the proliferation of cells was detected by CCK-8 and Edu assay. Finally, we analyzed the relationship between the expression of NAA50 and cell cycle related proteins.

**Results:** Pan-cancer analysis indicated that NAA50 was overexpressed in most cancers. And there was a significant correlation between NAA50 expression and the prognosis of cancer patients. In the meantime, NAA50 gene changes occur in a variety of tumors. Compared with normal tissues, the methylation level of NAA50 promoter increased in most cancer tissues. In addition, the results exhibited that in most cancers, NAA50 was significantly positively correlated with bone myeloid-derived suppressor cell (MDSC) infiltration and negatively correlated with T cell NK infiltration. Moreover, functional enrichment indicated that NAA50 regulates cell cycle and proliferation in LUAD. *In vitro* experiments testified that knockout of NAA50 could significantly inhibit the proliferation of LUAD.

**Conclusion:** NAA50 may be a potential biomarker and oncogene of pan-cancer, especially LUAD, which may promote the occurrence and development of tumors through different mechanisms. Furthermore, NAA50 was bound up with to immune cell infiltration in pan-cancer, meaning NAA50 may be an important therapeutic target for human cancers.

## Introduction

Nowadays, malignant tumor is developing into the main disease harming human health (Lin et al., 2022). There are numerous strategies to deal with cancers including chemotherapy, surgery, radiotherapy, immunotherapy, targeted therapy and combinational therapy (Najafi et al., 2021). Among them, immunotherapy adopts a different mechanism from traditional treatment, providing better choices for patients, and has become an important strategy for anti-cancer (Chen et al., 2021). The great success of immunotherapy makes the identification of immune related biomarkers an attractive field. However, in terms of treatment strategy, the overall survival rate (OS) of patients is still unsatisfactory due to the complexity and heterogeneity of tumorigenesis (Jia et al., 2022; Siegel et al., 2022). Therefore, it is extremely necessary to search new molecular targets to improve the treatment of cancer and predict the prognosis of patients with cancer.

Protein N-terminal acetylation is one of the most common covalent modifications in eukaryotes (Arnesen et al., 2009). This modification could affect many protein functions, including protein half-life, folding, localization, and complex formation, ultimately impacting cell and organism function (Aksnes et al., 2019; Koufaris and Kirmizis, 2020). Moreover, N-terminal acetylation of proteins is considered to be an irreversible process, which is catalyzed by N-terminal acetyltransferase (NAT) (Deng et al., 2020). Up to now, eight NATs (NatA-NatH) and their catalytic subunits (NAA10-NA80) have been known, and they are different in evolutionary protection, localization and target library (Koufaris and Kirmizis, 2020, 2021). Human cells encode catalytic subunits NAA10-NA60 and NAA80, as well as related auxiliary subunits, while NAA70 (NatG) is only found in plants (Aksnes et al., 2019). Among these catalytic subunits, N-Alpha-Acetyltransferase 50 (NAA50) has been reported to have acetyltransferase activity and is important for the separation of chromosomes (Evjenth et al., 2009; Deng et al., 2019). At the same time, NAA50 and NatA together constitute the NatE complex (Støve et al., 2016). Recently, people have paid more and more attention to NATs and their related biological functions, because new evidence revealed that NATs are related to major human diseases such as cancer. For instance, In Glioblastoma, NAA30 is involved in the regulation of gliogenesis and the p53 pathway (Mughal et al., 2015). NAA40 possesses potentially oncogenic functions in colorectal cancer and liver hepatocellular carcinoma (Demetriadou et al., 2019; Koufaris and Kirmizis, 2021). NAA10 is overexpressed in various types of cancer, affecting the overall survival rate and disease recurrence of cancer patients. (Yang et al., 2016; Kim et al., 2020). However, the functional and prognostic roles of NAA50 in cancers remain unknown and elusive.

In our research, we systematically analyzed the role of NAA50 in pan-cancer for the first time and verified it through relevant experiments. Firstly, we carried out a comprehensive analysis using specific databases to compare the expression and mutation of NAA50 in different types of tumors and adjacent normal tissues. Moreover, the potential prognostic value of NAA50 in pan-cancer was also evaluated. At the same time, we explored the potential signaling pathway of NAA50 involved in tumorigenesis and development, and studied the relationship between NAA50 and immune infiltration. Combined with bioinformatics analysis, we found that NAA50 was not only highly expressed in lung adenocarcinoma, but also closely related to poor prognosis, stage and immune infiltration of lung adenocarcinoma. In addition, signal pathways were identified by single-cell and enrichment analysis. Finally, we chose lung adenocarcinoma for *in vitro* experiments (CCK-8 and EDU experiments) to confirmed our bioinformatics results. In conclusion, we systematically and comprehensively verified the function, potential mechanism and relationship with immune infiltration of NAA50 gene in pan-cancer, especially in LUAD.

## Materials and methods

### Gene expression analysis

The TIMER2.0 database (http://timer.comp-genomics.org/) and Sangerbox3.0 (http://vip.sangerbox.com/home.html) were utilized to explore and prove the NAA50 in different types of tumors and adjacent normal tissues. And UCSC Genome Browser on Human (http://genome-asia.ucsc.edu/index.html) was used to explore Genomic View for NAA50 Gene. Additionally, the immunohistochemical (IHC) staining of the NAA50 in pan-cancer was studied by the Human Protein Atlas database (https://www.proteinatlas.org/). At the same time, the RNA expression and localization of NAA50 gene in different cell lines were compared.

### Diagnostic and prognostic analysis

In the present study, we analyzed the potential value of NAA50 in cancer prognosis *via* “Survival plots” and “Stage plots” modules of the GEPIA database. In addition, Cox analysis based on the SangerBox3.0 database was used for the overall survival of NAA50 in pan-cancer, and the outcome was shown by a forest plot.

### Mutation character and methylation analysis

The cBioPortal database (http://www.cbioportal.org/) was applied to analyze the mutation character of NAA50 in pan-cancers. “TCGA Pan Cancer Atlas Studies” was chosen for the cohort and entered “NAA50” in the “Query” module. So as to find the alteration sites, types, and numbers of NAA50 in the “cancer type summary” and “mutation” modules.

In the UALCAN database (http://ualcan.path.uab.edu), the “TCGA gene analysis” function was used to explore the difference in NAA50 DNA promoter methylation levels between tumor tissues and normal tissues. The DNA promoter methylation levels of NAA50 in 8 cancers were obtained.

### Immune cell infiltration analysis

Firstly, TISIDB (http://cis.hku.hk/TISIDB/index.php) database was applied to comprehend the distribution of NAA50 expression across immune subtypes. In addition, it was applied to infer the relationship between NAA50 and major histocompatibility complexes (MHCs) and Chemokines. Finally, the relationship between infiltrates level of immune cells estimation value and NAA50 expression was evaluated by the “GENE” module of the TIMER2.0 database.

### The function and enrichment analysis

CancerSEA website (biocc.hrbmu.edu.cn/CancerSEA/home.jsp) was applied to explore the Single-cell sequence data relationship between NAA50 and cancer functional states through the “correlation plot” module. Subsequently, co-expression analysis of genes belonging to NAA50 was performed using LinkedOmics (www.linkedomics.org/login.php). The “TCGA_LUAD” cohort and the “HiSeq RNA” platform were the sample cohort for our analysis. The correlation of co-expression genes was checked by the Pearson test.

### Cell cultures and transfection

LUAD cell lines H1299 and PC9 were from Shanghai Academy of Sciences. The medium was used the PPMI-1640 plus 10% fetal bovine serum (FBS; Gibco, United States) and cells were incubated at 37°C with 5% CO_2_. The siRNA for NAA50 mRNA was produced by GenePharma. The siRNA template sequences in our study were applied as following: siNAA50-1, sense 5′-CAC​CAC​ACA​AUA​UUA​AAC​ATT-3′, antisense 5′-UGU​UUA​AUA​UUG​UGU​GGU​GTT-3′, siNAA50-2, sense 5′-CUU​GCC​UAU​UUC​AAU​GAU​ATT-3′, antisense 5′-UAU​CAU​UGA​AAU​AGG​CAA​GTT -3′. According to the manufacturer’s protocol, the H1299 cells and PC9 cells were transfected with miRNAs using the jetPRIME transfection reagent (Polyplus-transfection, Illkirch, France).

### Western blotting

The total protein was collected in RIPA buffer (Beyotime, Shanghai, China), after that centrifuged at 4°C for 10 min. Then we would collect the supernatant and calculate the protein concentration using BCA kit (Beyotime, Shanghai, China). The protein samples were electrophoretized on sodium dodecyl sulfate-polyacrylamide (SDS-PAGE) gel, after that transferred to a polyvinylidene fluoride (PVDF) membrane. At room temperature 5% milk powder was sealed for 2 h and incubated with primary antibody overnight at 4°C. The antibodies used were as follows: GAPDH (AC033; ABclonal); NAA50 (ER2001-66; HUABIO). On the next day, after washing three times with TBST, they were incubated with secondary antibodies for 1 h at room temperature. The signal is then detected by enhanced chemiluminescence as recommended by the manufacturer.

### EdU assay and CCK-8 assay

For EdU (RiboBio, Guangzhou, China) analysis, cells were first cultured in a medium containing 10 mol/L EdU for 2 h. After removing the medium containing EdU, 4% paraformaldehyde was applied to fix with the cells at room temperature for 30 min. Discard paraformaldehyde, add glycine and incubate for 5 min. 1xApollo reaction solution and 1xHoechst reaction solution diluted by 100 times were added and incubated for 30 min under dark conditions. Imaging was performed using a fluorescence microscope.

The transduced cells were seeded into 96-well plates (2,000 cells/well). Subsequently, 10 μl of the CCK-8 (APExBIO, #K1018) was mixed to every well. The absorbance at 450 nm was measured after incubating the cells for 2 h in dark.

### Statistical analysis

The data were expressed as means ± standard deviation (SD). GraphPad Prism 8 software was used for all statistical analyzes. The correlation analysis was estimated with Spearman’s test. The differences among two groups were detected using *t*-test. Meanwhile, the significance of differences among more than 2 groups was assessed using Two-way ANOVA analysis. It is important that *p* < 0.05 was considered to be significant.

## Results

### The abnormal expression of N-alpha-acetyltransferase in human pan-cancers

**FIGURE 1:**
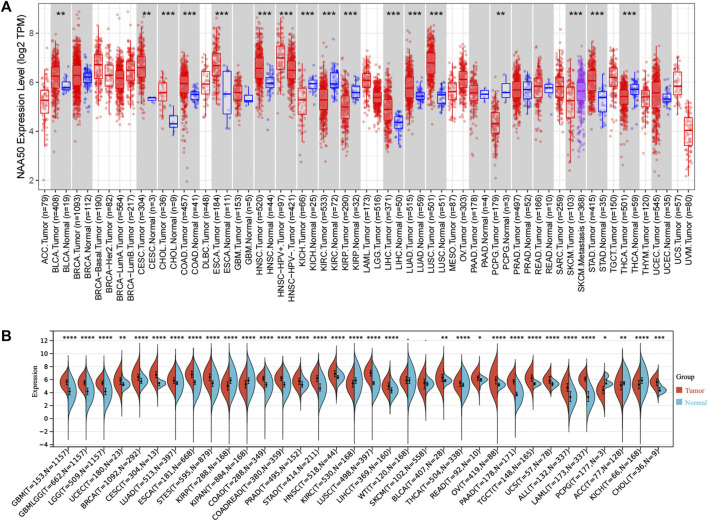
The NAA50 expression profiles in pan-cancer. **(A)** Pan-cancer analysis of NAA50 in TIMER2.0 database. **(B)** The expression analysis of NAA50 in SangerBox3.0 database.

Firstly, in order to explore the expression of NAA50 in human cancer, we used the TIMER2.0 database to detect the expression of NAA50. The results obtained showed that NAA50 expression levels increased in various cancer tissues, including Bladder Urothelial Carcinoma (BLCA), Cervical squamous cell carcinoma and endocervical adenocarcinoma (CESC), Colon adenocarcinoma (COAD), Esophageal carcinoma (ESCA), Head and Neck squamous cell carcinoma (HNSC), Liver hepatocellular carcinoma (LIHC), Lung adenocarcinoma (LUAD), Lung squamous cell carcinoma (LUSC), Stomach adenocarcinoma (STAD) (Figure 1A). Then, SangerBox3.0 online database was used to analyze the NAA50 expression in TCGA and GTEx datasets, significantly overexpressed of NAA50 were observed in glioblastoma multiforme (GBM), Glioma (GBMLGG), Brain Lower Grade Glioma (LGG), Uterine Corpus Endometrial Carcinoma (UCEC), Breast invasive carcinoma (BRCA), CESC, LUAD, ESCA, Stomach and Esophageal carcinoma (STES), Colon adenocarcinoma (COAD), Colon adenocarcinoma/Rectum adenocarcinoma Esophageal carcinoma (COADREAD), Prostate adenocarcinoma (PRAD), STAD, HNSC, LUSC, LIHC, BLCA, Thyroid carcinoma (THCA), Rectum adenocarcinoma (READ), Ovarian serous cystadenocarcinoma (OV), Pancreatic adenocarcinoma (PAAD), Testicular Germ Cell Tumors (TGCT), Uterine Carcinosarcoma (UCS), Acute Lymphoblastic Leukemia (ALL) and Acute Myeloid Leukemia (LAML), Cholangiocarcinoma (CHOL) (Figure 1B). However, combining the two analyses, it can be concluded that NAA50 was downregulated in Kidney Chromophobe (KICH), Kidney renal clear cell carcinoma (KIRC), Kidney renal papillary cell carcinoma (KIRP) and Pheochromocytoma and Paraganglioma (PCPG).

### Analysis of N-alpha-acetyltransferase 50 gene expression and location

**FIGURE 2:**
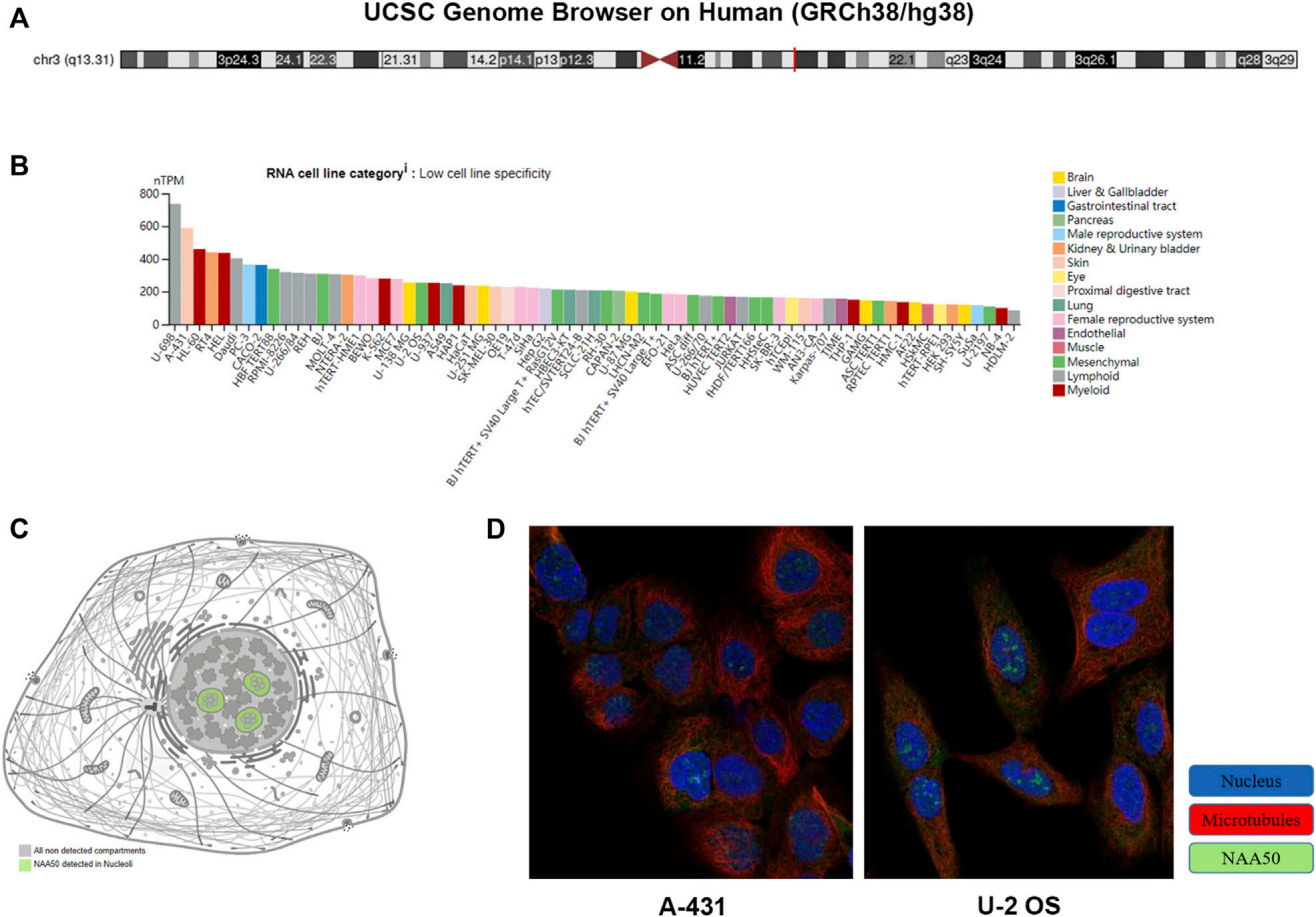
The expression and location analysis of NAA50. **(A)** Genomic location of human NAA50 gene. **(B)** The RNA expression patterns of NAA50 in various cell lines. **(C)** The NAA50 protein can be secreted. **(D)** NAA50 protein is localized to the nucleoli of A-431 and U-2 OS cells.

**FIGURE 3:**
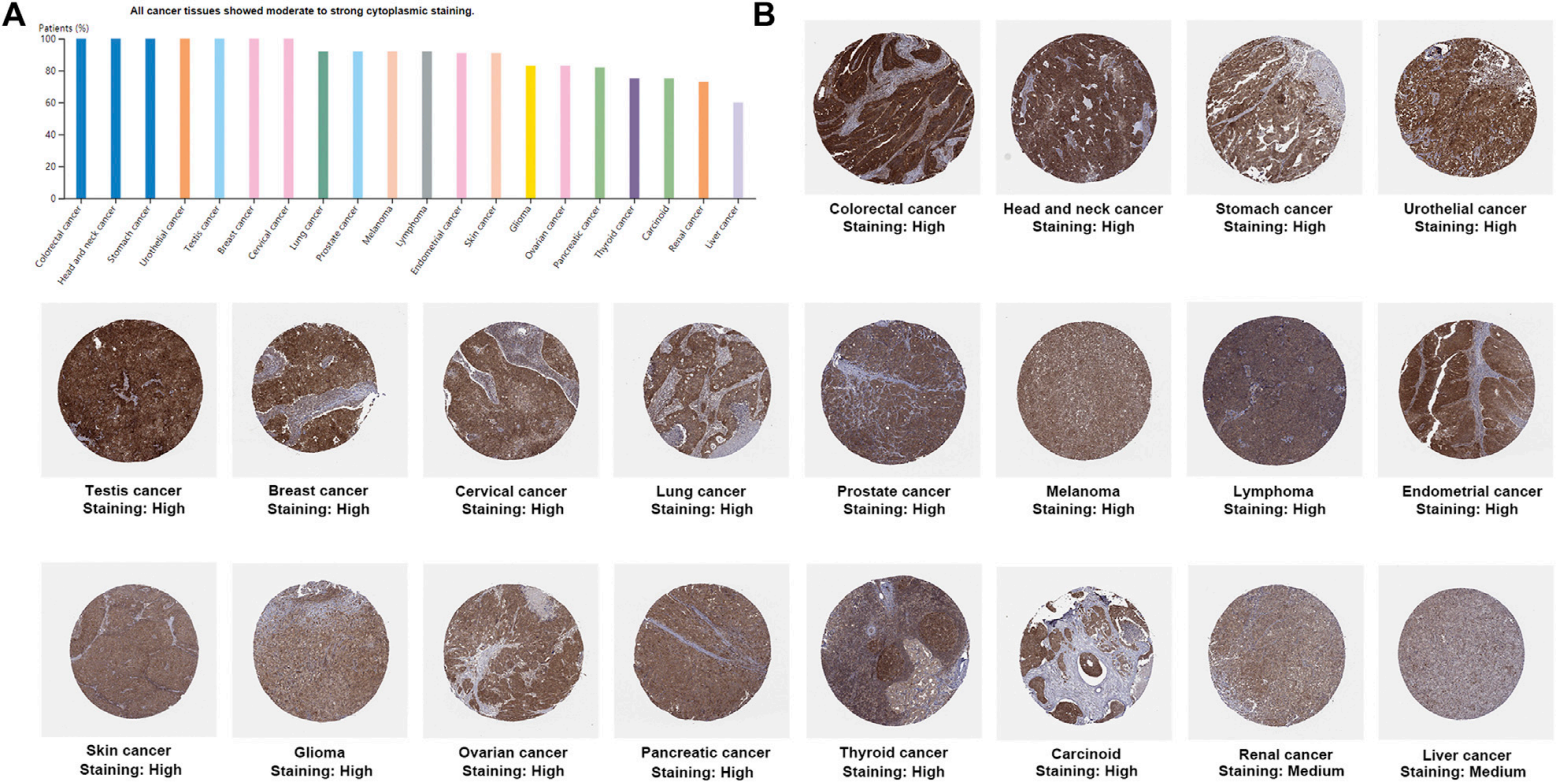
The protein expression levels NAA50 in cancer tissues. **(A)** The percentage of cancer patients (maximum 12 patients) with high and medium protein expression level. **(B)** Immunohistochemistry results of NAA50 protein assessed in pan-cancer.

UCSC Genome Browser on Human (GRCh38/hg38) was used to explore Genomic View for NAA50 Gene (Figure 2A). And we performed an analysis of the RNA expression patterns of NAA50 in various cell lines (Figure 2B). As we can see, the expression of NAA50 was at high levels in U-698, A-431 and HL-60. Additionally, according to the summary of RNA expression and protein localization based on data generated within the Human Protein Atlas project, the NAA50 protein was localized to the Nucleoli (supported) (Figure 2C). Next, NAA50 protein was localized to the nucleoli of A-431 cell and U-2 OS cell, DAPI for the nucleus in blue, the NAA50 protein staining was shown in green (Figure 2D). Surprisingly, in the Human Protein Atlas database, the IHC results of NAA50 demonstrated that all cancer tissues exhibited moderate to strong cytoplasmic staining (Figure 3A). Meanwhile, Figure 3B manifested that the expression level of total NAA50 protein was significantly increased in Colorectal cancer, Head and neck cancer, Stomach cancer, Urothelial cancer, Testis cancer, Breast cancer, Cervical cancer, Lung cancer, Prostate cancer, Melanoma, Lymphoma, Endometrial cancer, Skin cancer, Glioma, Ovarian cancer, Pancreatic cancer, Thyroid cancer, Carcinoid. Renal cancer and Liver cancer. Therefore, the results of these protein levels indicated that NAA50 was closely related to pan-cancer.

### The prognostic value of N-alpha-acetyltransferase 50 in pan-cancer

**FIGURE 4:**
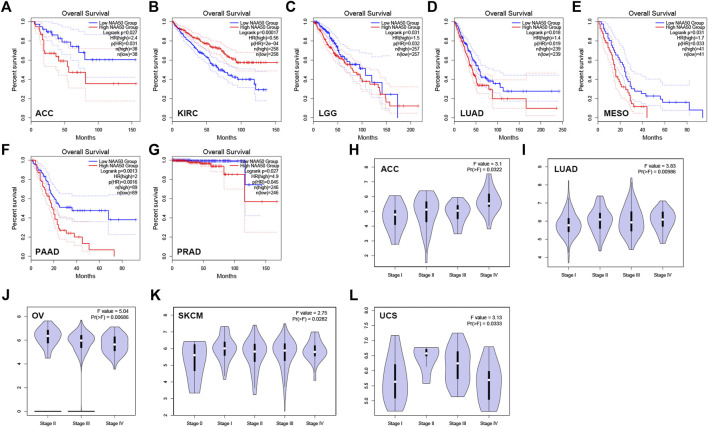
The correlation between the expression of NAA50 and prognosis of cancers by using GEPIA database. **(A–G)** The correlation between NAA50 expression and Overall survival. **(H–L)** The correlation between NAA50 expression and cancer stage.

**FIGURE 5:**
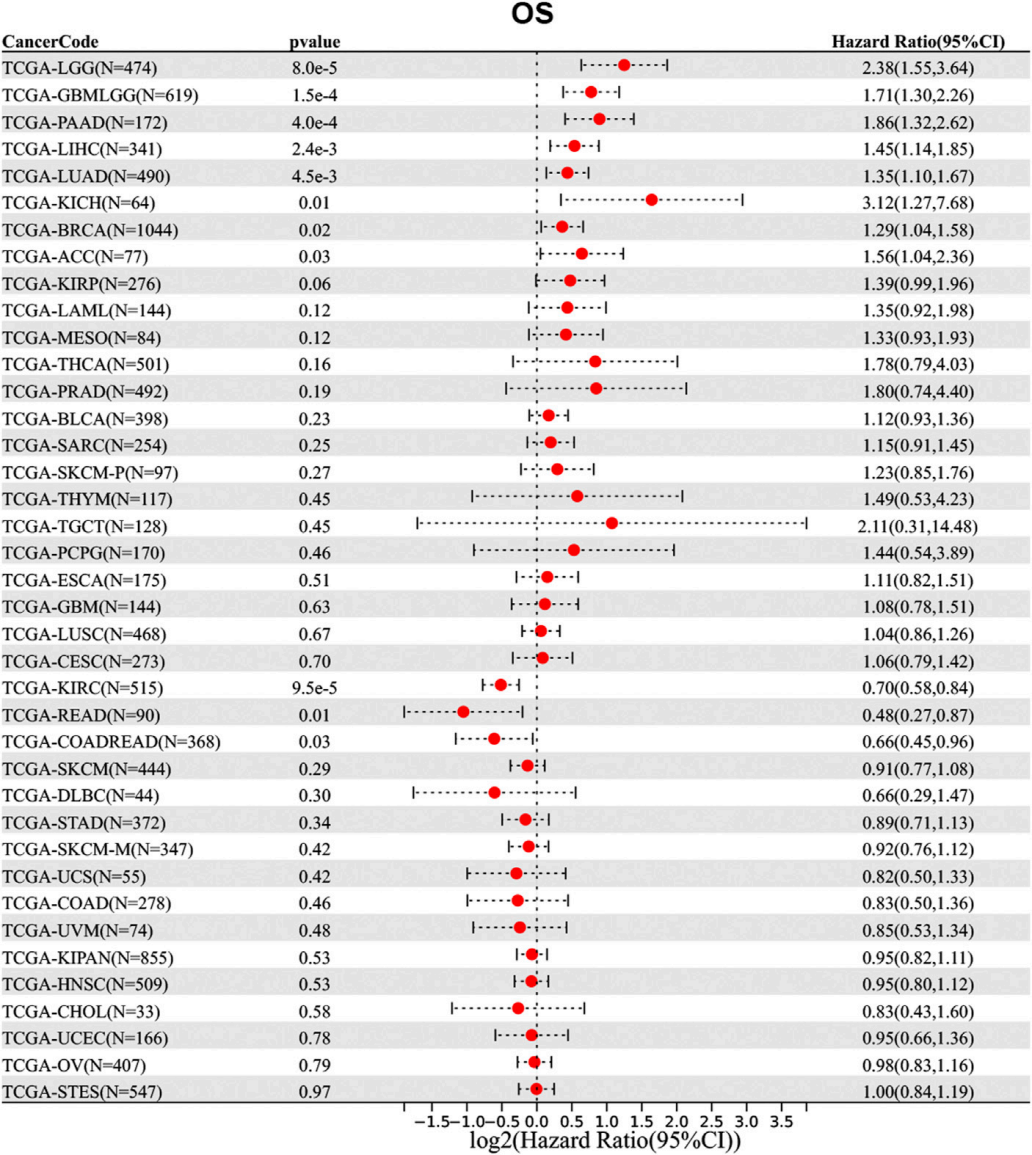
Correlation between the NAA50 expression and survival prognosis of cancers in TCGA datasets.

Then, we analyzed the diagnostic value of NAA50 in various cancers using GEPIA database. As shown in Figures 4A–G, increased expression of NAA50 was linked to poor prognosis in ACC (*p* = 0.027), LGG (*p* = 0.031), LUAD (*p* = 0.018), MESO (*p* = 0.031), PAAD (*p* = 0.0013) and PRAD (*p* = 0.027). But the increased expression of NAA50 was associated with a good prognosis in KIRC (*p* = 0.00017). Meanwhile, we analyzed the association between NAA50 and the stages of pan-cancers. NAA50 was associated with the stage of ACC (*p* = 0.0322), LUAD (*p* = 0.00986), OV (*p* = 0.00686), SKCM (*p* = 0.0282), UCS (*p* = 0.0333) (Figures 4H–L). Furthermore, we acquire a more comprehensive prognostic value of NAA50 by using the Cox analysis in the SangerBox3.0 database (Figure 5). The results revealed that the high expression of NAA50 was significantly correlated with poor prognosis in 8 tumor types, which were TCGA-GBMLGG [N = 619, *p* = 1.5e-4, HR = 1.71 (1.30, 2.26)], TCGA-LGG [N = 474, *p* = 8.0e-5, HR = 2.38 (1.55, 3.64)], TCGA-LUAD [N = 490, *p* = 4.5e-3, HR = 1.35 (1.10, 1.67)], TCGA-BRCA [N = 1,044, *p* = 0.02, HR = 1.29 (1.04, 1.58)], TCGA-LIHC [N = 341, *p* = 2.4e-3, HR = 1.45 (1.14, 1.85)], TCGA-PAAD [N = 172, *p* = 4.0e-4, HR = 1.86 (1.32, 2.62)], TCGA-ACC [N = 77, *p* = 0.03, HR = 1.56 (1.04, 2.36)], TCGA-KICH [N = 64, *p* = 0.01, HR = 3.12 (1.27, 7.68)]. And low expression in 3 tumor types TCGA-KIRC [N = 515, *p* = 9.5e-5, HR = 0.70 (0.58, 0.84)], TCGA-COADREAD [N = 368, *p* = 0.03, HR = 0.66 (0.45, 0.96)] and TCGA-READ [N = 90, *p* = 0.01, HR = 0.48 (0.27, 0.87)] with poor prognosis.

### Analysis of N-alpha-acetyltransferase 50 mutation and methylation level in pan-cancer

**FIGURE 6:**
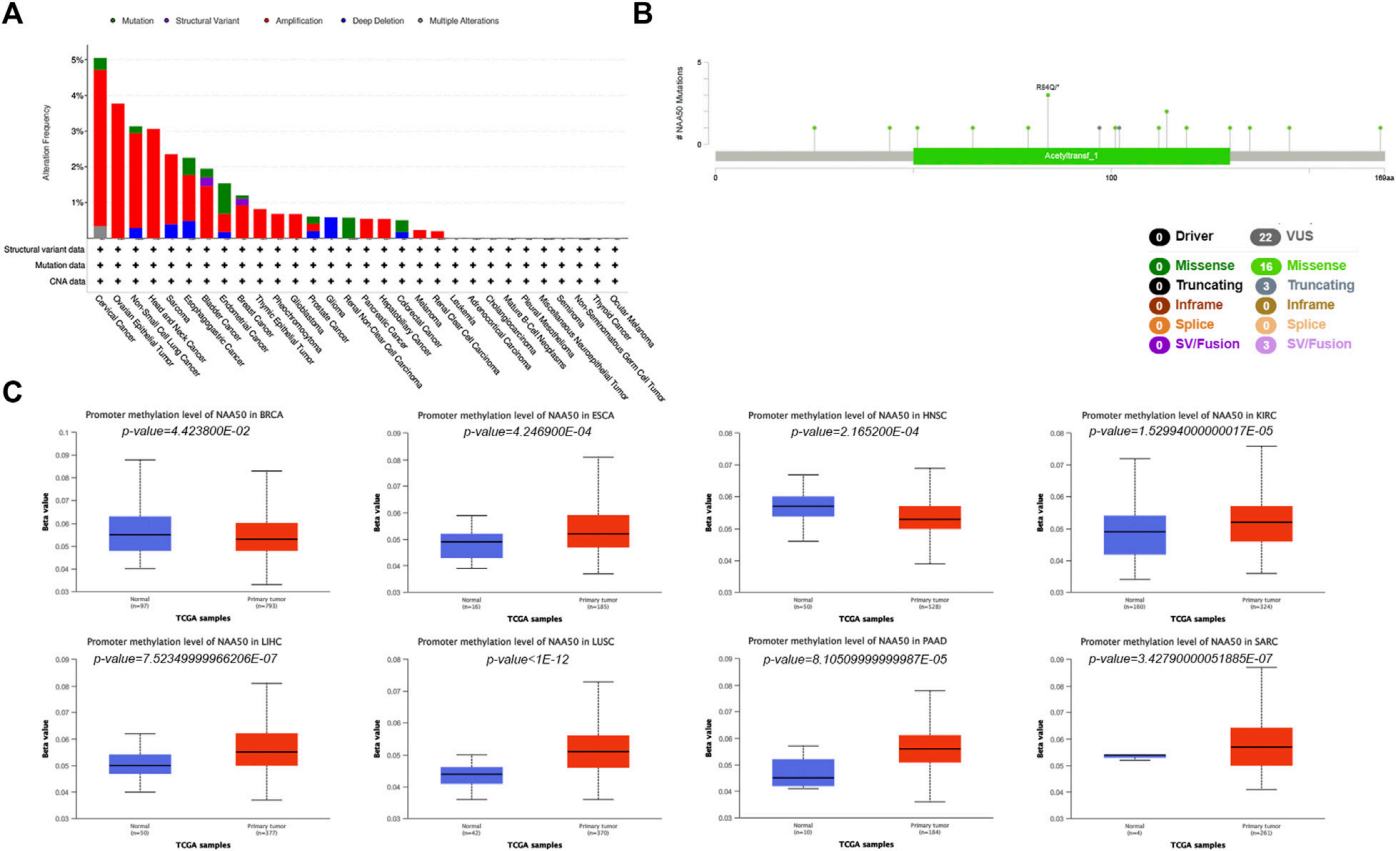
Mutation and Methylation feature of NAA50 in different cancers. **(A)** Alteration frequency with the mutation type of NAA50 in pan-cancer. **(B)** The subtypes and distributions of NAA50 somatic mutations. **(C)** The differential DNA methylation level of NAA50 promoter in pan-cancer.

In order to more systematically clarify the mutation characteristics of NAA50 in tumor progression, we analyzed the NAA50 alteration status of the TCGA cohorts through the online database cBioPortal. Genetic alterations in NAA50 were dominated by “Amplification” types, which were observed in almost TCGA cancer cases, except Glioma, Renal Non-Clear Cell Carcinoma and Colorectal Cancer. In Glioma, the genetic changes are completely “Deep Deletion”, while in Renal Non-Clear Cell Carcinoma, they are completely mutant “Mutation” (Figure 6A). To great understand the mutational map of NAA50 in different cancer types, our investigation displayed the NAA50 alteration sites, types, and numbers are further presented in Figure 6B. Hence, we next used the UALCAN database to explore the level of NAA50 methylation in pan-cancer and its corresponding tissues. Compared with adjacent normal tissues, the methylation level of NAA50 promoter in BRCA and HNSC decreased. In contrast, the methylation level of NAA50 promoter in ESCA, KIRC, LIHC, LUSC, PAAD and SARC was higher than that in their adjacent normal tissues (Figure 6C).

### Pan-cancer analysis of N-alpha-acetyltransferase 50 expression and immune cell infiltration

**FIGURE 7:**
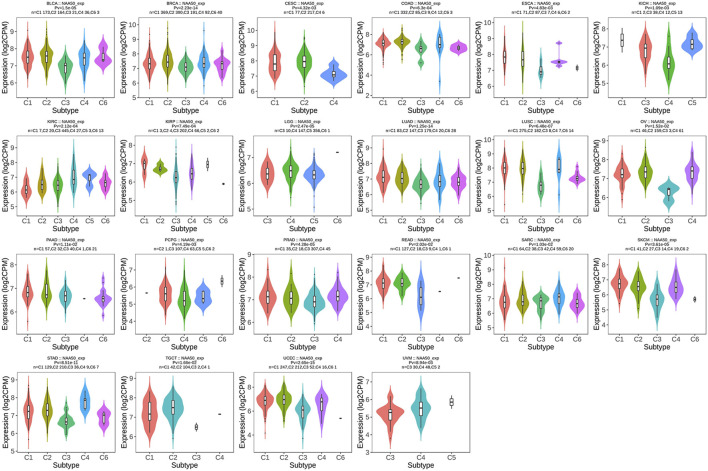
Associations between the NAA50 expression and immune subtypes across human cancers.

**FIGURE 8:**
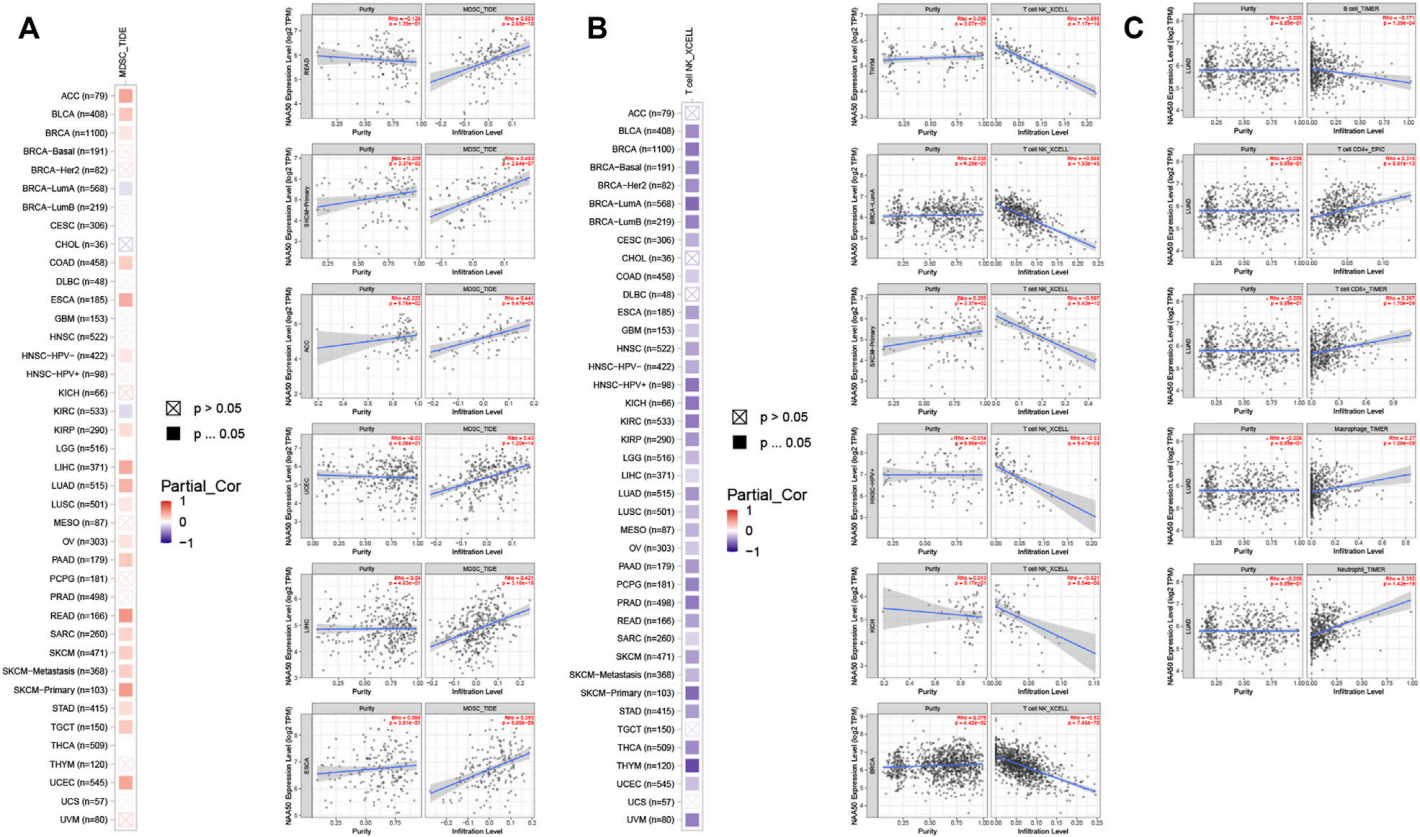
Immune cell infiltration analysis of NAA50 in the TIMER2.0 database. **(A)** The NAA50 expression was positively associated with MDSC infiltration in pan-cancer. **(B)** The NAA50 expression was negatively associated with NKT cell infiltration in pan-cancer. **(C)** The relationship between the expression of NAA50 and the infiltration level of B cells, CD4+ T cells, CD8+ T cells, macrophages and neutrophils in LUAD.

**FIGURE 9:**
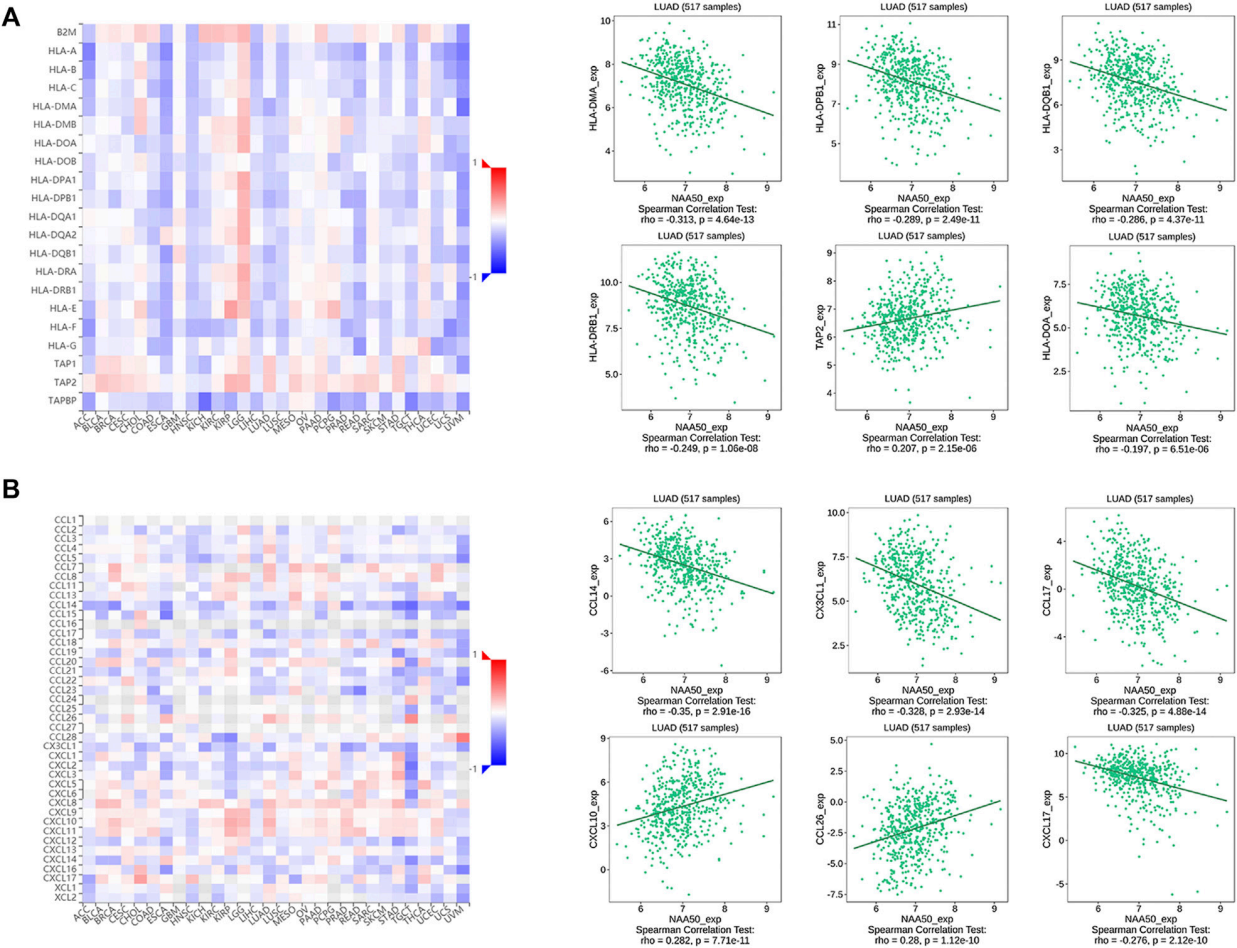
Correlation analysis between the NAA50 expression and major histocompatibility complexes (MHCs) and chemokines in the TISIDB database. **(A)** MHCs. **(B)** Chemokines.

Since the immune microenvironment plays a critical role in tumorigenesis and cancer progression, we further evaluated the associations between NAA50 expression and immune subtypes across human cancers based on the TISIDB database. The immune subtypes included: C1 (wound healing), C2 (IFN-gamma dominant), C3 (inflammatory), C4 (lymphocyte depleted), C5 (immunologically quiet) and C6 (TGF-b dominant). As we can see that the expression of NAA50 was significantly correlated with the immune subtypes in BLCA, BRCA, CESC, COAD, ESCA, KICH, KIRC, KIRP, LGG, LUAD, LUSC, OV, PAAD, PCPG, PRAD, READ, SARC, SKCM, STAD, TGCT, UCEC and UVM (Figure 7). Then, we discussed the potential role of NAA50 in immune cell infiltration based on TIMER2.0 database. The following results manifested that in most tumors, the infiltration level of myeloid-derived suppressor cells (MDSC) was positively bound up with the expression of NAA50 (Figure 8A). The top six tumors were READ (rho = 0.503, *p* = 2.68e-10), SKCM-Primary (rho = 0.483, *p* = 2.64e-07), ACC (rho = 0.441, *p* = 9.47e-05), UCEC (rho = 0.430, *p* = 1.20e-14), LIHC (rho = 0.421, *p* = 3.16e-16) and ESCA (rho = 0.393, *p* = 5.00e-08). At the same time, our study found that the infiltration level of T cell NK in most tumors was negatively correlated with the expression of NAA50 (Figure 8B). THYM (rho = −0.695, *p* = 7.17e-18), BRCA-LumA (rho = −0.568, *p* = 1.50e-45), SKCM-Primary (rho = −0.567, *p* = 5.40e-10), HNSC-HPV+ (rho = −0.530, *p* = 9.47e-08), KICH (rho = −0.521, *p* = 8.54e-06) and BRCA (rho = −0.520, *p* = 7.46e-70) were in the top six. After that, we studied the role of NAA50 in LUAD immune cell infiltration. There was a negative association between NAA50 and the infiltration level of B cells (rho = −0.171, *p* = 1.39e-04), and there was a positive association between NAA50 and the infiltration level of CD4+ T cells (rho = 0.315, *p* = 8.81e-13), CD8+ T cells (rho = 0.267, *p* = 1.70e-09), Macrophage (rho = 0.27, *p* = 1.08e-09) and Neutrophil (rho = 392, *p* = 1.42e-19) (Figure 8C). In the following study, the association between NAA50 and MHCs and Chemokines was further analyzed through the TISIDB database. Our results indicated that the expression of NAA50 was negatively correlated with most MHCs. The top 6 MHCs were HLA-DMA (rho = −0.313, *p* = 4.64e-13), HLA-DPB1 (rho = −0.289, *p* = 2.49e-11), HLA-DQB1 (rho = −0.286, *p* = 4.37e-11), HLA-DRB1 (rho = −0.249, *p* = 1.06e-08), TAP2 (rho = 0.207, *p* = 2.15e-06), and HLA-DOA (rho = −0.197, *p* = 6.51e-06) in LUAD (Figure 9A). Figure 9B displayed the NAA50 was negatively associated with most Chemokines, and the top 6 in LUAD were CCL14 (rho = −0.35, *p* = 2.91e-16), CX3CL1 (rho = −0.328, *p* = 2.93e-14), CCL17 (rho = −0.325, *p* = 4.88e-14), CXCL10 (rho = 0.282, *p* = 7.71e-11), CCL26 (rho = 0.28, *p* = 1.12e-10), and CXCL17 (rho = −0.276, *p* = 2.12e-10).

### The function analysis of N-alpha-acetyltransferase 50 in cancers

**FIGURE 10:**
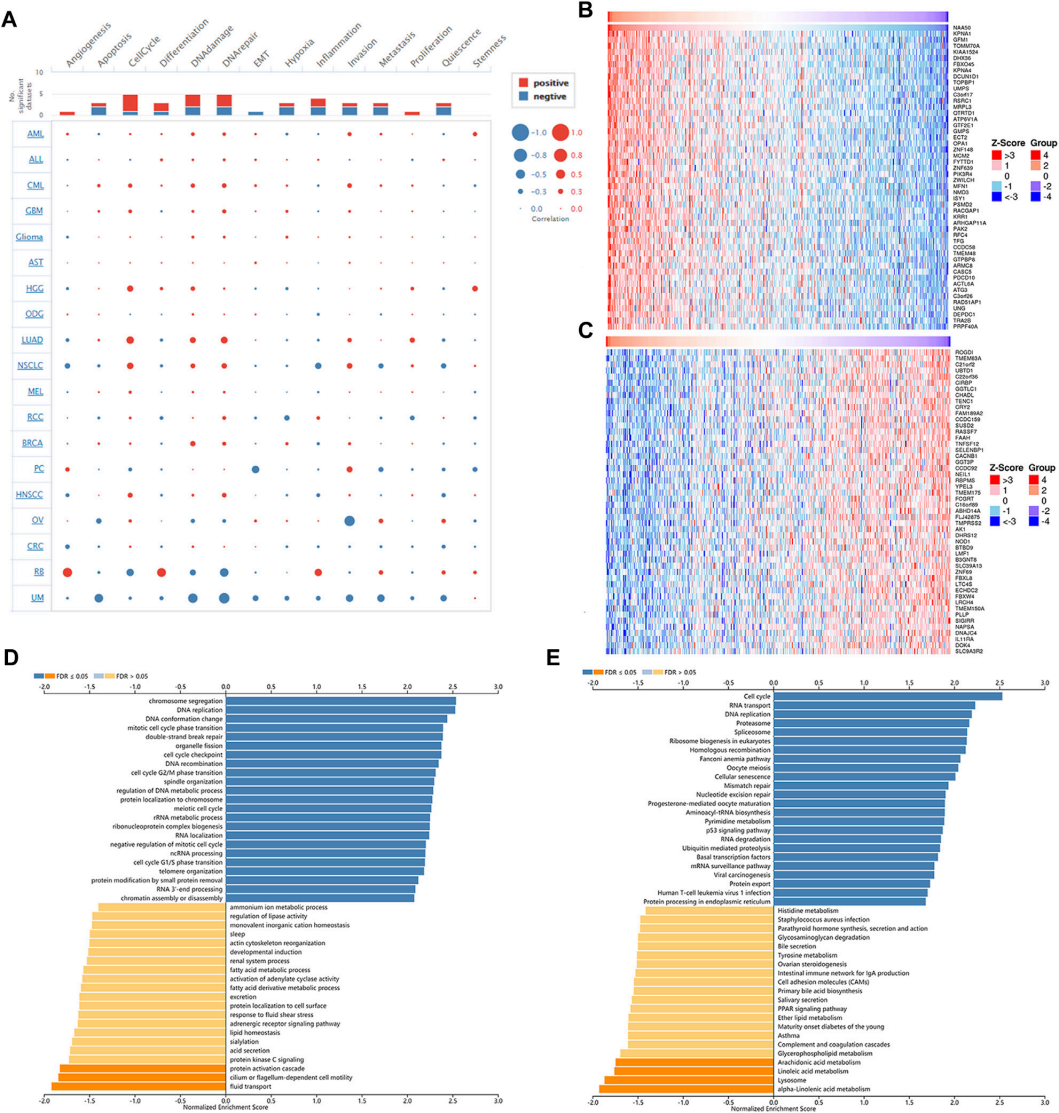
Single-Cell Analysis of NAA50 and NAA50-related gene enrichment analysis. **(A)** The correlation between NAA50 expression and cancer functional states in pan-cancer. **(B)** The top 50 genes positively correlated to NAA50. **(C)** The top 50 genes negatively correlated to NAA50. **(D)** GO (biological function) analysis of NAA50 co-expression genes in the LUAD cohort. **(E)** KEGG analysis of NAA50 co-expression genes in the LUAD cohort.

Next, single-cell sequence data of CancerSEA was used to further analyze the correlation between NAA50 and pan-cancer functional states. In most tumors, NAA50 was positively correlated with cell cycle and invasion (Figure 10A). It was worth noting that NAA50 plays a particularly prominent role in LUAD. Combined with prognosis analysis, we decided to further explore the biological function of NAA50 in LUAD through LinkedOmics database. Figures 10B,C indicated the top 50 genes that were positively and negatively correlated with NAA50. In addition, GO analysis (biological function) showed that NAA50 was mainly involved in chromosome segregation, DNA replication, DNA conformation change, mitotic cell cycle phase transition, double-strand break repair, organelle fission, cell cycle checkpoint, DNA recombination, etc. (Figure 10D). Moreover, KEGG analysis showed enrichment of Cell cycle, RNA transport, DNA replication, Fanconi anemia pathway, p53 signaling pathway, mRNA surveillance pathway, Human T cell leukemia virus 1 infection and so on (Figure 10E).

### Biological functions of N-alpha-acetyltransferase 50 in human lung adenocarcinoma cells

**FIGURE 11:**
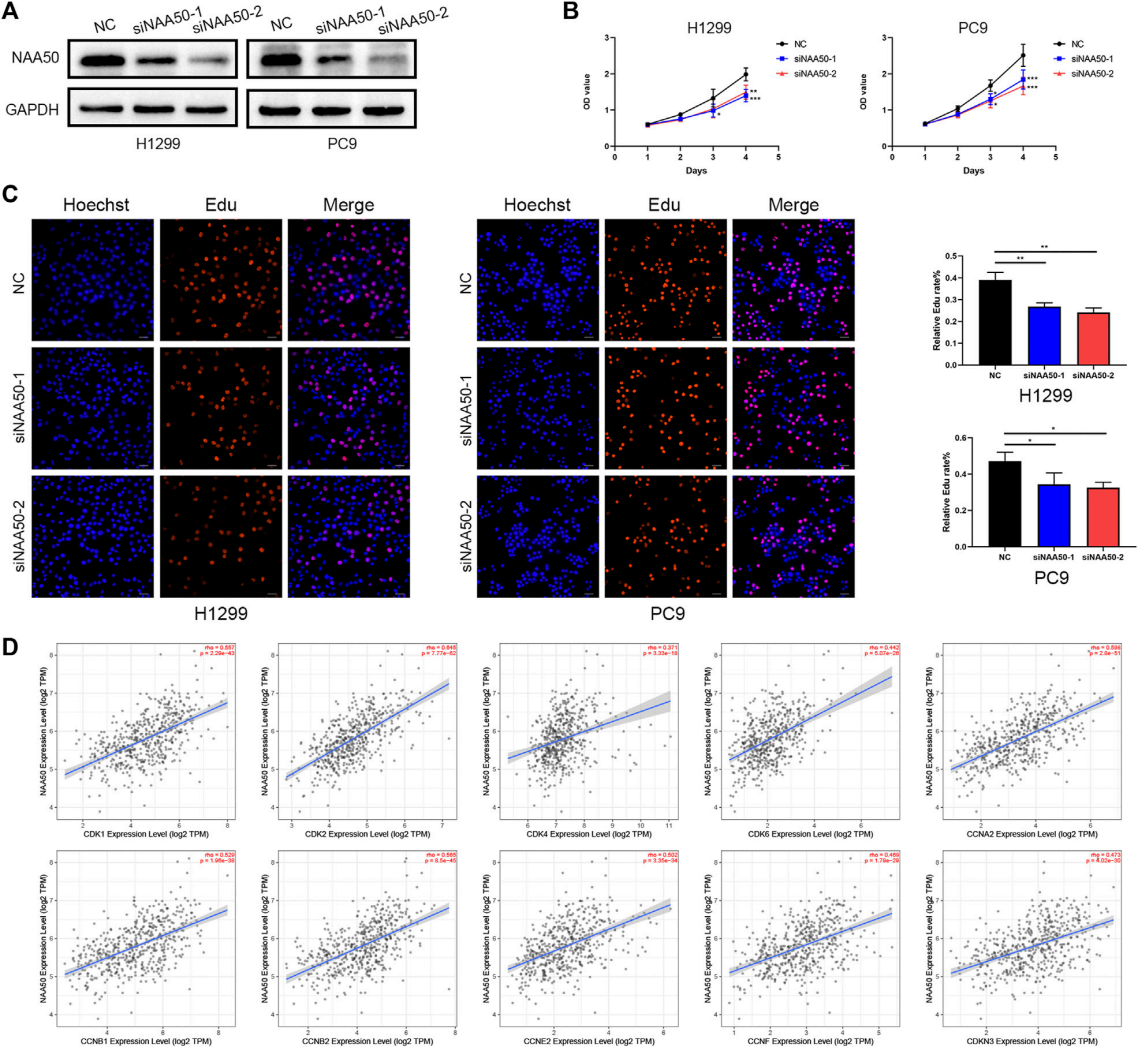
Knockdown of NAA50 significantly suppressed proliferation and cell cycle associated protein analysis. **(A)** The expression of NAA50 after siRNA transfection was detected by Western blot in H1299 cell and PC9 cell lines. **(B)** CCK-8 assay was used to detect the effect of NAA50 downregulation on proliferation in H1299 cell and PC9 cell lines. **(C)** EDU assay was used to detect the effect of NAA50 downregulation on proliferation in H1299 cell and PC9 cell lines. The data were expressed as means ± standard deviation (SD). *p < 0.05, **p < 0.01, ***p < 0.001. **(D)** The correlation between the NAA50 expression and CDK1, CDK2, CDK4, CDK6, CCNA2, CCNB1, CCNB2, CCNE2, CCNF, and CDKN3 in the TIMER2.0 database.

Subsequently, in H1299 and PC9 cell lines, we knocked down NAA50 to verify the role of NAA50 in lung adenocarcinoma. The knockout effect was detected using western blot (Figure 11A). Next, CCK-8 assay was carried out to evaluate cell proliferation. The results suggest a significant inhibition of proliferation rates in the tumor cells with NAA50 downregulation (Figure 11B). And the results of EdU Assay indicated that the proliferation ability of H1299 cells and PC9 cells were significantly inhibited when NAA50 was silenced (Figure 11C). Meanwhile, we found that the expression of NAA50 was remarkably correlated with the cell cycle by the biological pathway analysis of LUAD. Therefore, we further investigated the association between NAA50 and CDK family, CDKI family, and Cyclin family expression in TIMER2.0 database. The result is just as we expected, the NAA50 expression was correlated with the expression of CDK1 (rho = 0.557, *p* = 2.29e-43), CDK2 (rho = 0.645, *p* = 7.77e-62), CDK4 (rho = 0.371, *p* = 3.33e-18), CDK6 (rho = 0.442, *p* = 5.07e-26), CCNA2 (rho = 0.598, *p* = 2.8e-51), CCNB1 (rho = 0.529, *p* = 1.96e-38), CCNB2 (rho = 0.565, *p* = 8.5e-45), CCNE2 (rho = 0.502, *p* = 3.35e-34), CCNF (rho = 0.469, *p* = 1.79e-29) and CDKN3 (rho = 0.473, *p* = 4.02e-30) (Figure 11D). Taken together, NAA50 increased the proliferation and was correlated with the cell cycle in LUAD.

## Discussion

The role of human N-terminal acetyltransferase (NAT) in cancer has attracted more and more attention. Manipulation of NATs in cancer cells can target multiple pathways leading to cell cycle arrest, apoptosis or autophagy (Kalvik and Arnesen, 2013; Grunwald et al., 2020). However, the potential role of NAA50 as a NatE complex in cancer was worthwhile to be disclosed. Our study found that NAA50 was remarkably upregulated in a variety of cancer tissues, and all cancer tissues exhibited moderate to strong cytoplasmic staining. At the same time, survival analysis exhibited that increased expression of NAA50 was linked to poor prognosis in ACC, LGG, LUAD, MESO, PAAD and PRAD. And we found that NAA50 was closely related to cancer stage in ACC, LUAD, OV, SKCM, and UCS. In addition, COX regression analyses suggested that elevated NAA50 expression may lead to shorter OS in GBMLGG, LGG, LUAD, BRCA, LIHC, PAAD, ACC and KICH. Notably, these results particularly revealed NAA50 as a carcinogenic indicator of LUAD prognosis, regardless of the prognostic algorithm. Next, we further carried out the single-cell analysis and enrichment analysis, the results identified that NAA50 was significantly enriched in the cell cycle pathway and was closely related to the progression of LUAD. This indicated that NAA50 plays a crucial role in LUAD. In addition, *in vitro* experiments verified the role of NAA50 in LUAD.

DNA methylation is the production of 5-methylcytosine by adding a methyl group to the fifth carbon atom of cytosine in DNA sequence under the catalysis of DNMT (Liang et al., 2021). The DNA methylation process of specific genes plays different biological roles in cancer. For example, DNA methylation can be used as an important marker of drug efficacy, 5-year survival rate and lung cancer recurrence (Liang et al., 2021). In addition, DNA methylation is closely related to the immune response of various cancers (Jung et al., 2019). In this study, we evaluated the methylation of NAA50 in pan-cancers. Results showed that the methylation level of NAA50 promoter in ESCA, KIRC, LIHC, LUSC, PAAD and SARC was higher than that in their adjacent normal tissues; while decreased in BRCA and HNSC. In short, DNA methylation analysis of NAA50 may provide a new understanding of the prognosis of NAA50 in pan-cancer.

In the past, more and more studies have testified that the prognosis of cancer patients was closely interrelated to the tumor microenvironment (TME) (Liu et al., 2017; Chen et al., 2020). Immune responses in the tumor microenvironment are also believed to play a huge role in the progression of cancer and response to treatment (Song et al., 2020; Ugolini et al., 2022). And immune infiltrating cells in TME are closely related to the improvement of diagnosis and treatment (Liu et al., 2021; Feng et al., 2022). Considering that the up regulation of NAA50 expression level in pan-cancer was related to short OS, we speculate that NAA50 may participate in tumor immune response. Therefore, we use TIMER 2.0 database to further verify the relationship between NAA50 and immune infiltration. Our study found that the expression of NAA50 was positively correlated with the majority of MDSC infiltration and negatively correlated with the majority of T cell NK infiltration. As for MDSC, it can make use of signaling pathways and multiple effector molecules to regulate immune suppression, including depletion of necessary amino acids, reactive oxygen species (ROS), PD-L1 expression and so on (Gabrilovich, 2017). At the same time, the upregulation expression of MDSC has been found in lymphoid tissue, peripheral blood and tumor sites and significantly bound up with poor prognosis in patients with multiple cancers (Li et al., 2021). T cell NK, a specialized subset of lipid-reactive T lymphocytes, produces direct and indirect roles in anti-tumor immunity and immunosurveillance and infiltration of it is associated with a great prognosis in some cancers (Nelson et al., 2021). Summarizing our results, we can infer that the up-regulated expression of NAA50 in pan carcinoma was related to poor prognosis, which may play a role by possibly recruiting MDSC and inhibiting T cell NK cell infiltration. In short, our study may provide new insights into the treatment of NAA50 and a direction for new immunotherapy approaches.

The cell cycle is a process, which enables cell growth, duplication of genetic material, and cell division (Suski et al., 2021). New research demonstrated that cancer cell cycle changes greatly, involving the etiology, progression and therapeutic intervention of cancer (Witkiewicz et al., 2022). The cyclin/cyclin-dependent kinase (CDK), essential for driving each cell cycle phase, is aberrant in numerous human cancers (Malumbres and Barbacid, 2009; Comstock et al., 2013). For instance, the binding of CDK2 to cyclin E or cyclin A can promote the progression of cell cycle G1/S/G2 (Coverley et al., 2002). Simultaneously, CDK4/CDK6 preferentially interact with cyclins to produce active complexes. And the catalytic activity of CDK4/6 is considered to be the key to initiate the G1/S phase of the cell cycle (Knudsen et al., 2022). In the meantime, the knockdown of CCNB2 blocked G2/M transition in breast cancer cell cycle and increased the number of apoptotic cells (Aljohani et al., 2022). Through online analysis, we found that the expression of NAA50 was closely and positively related to cell cycle related proteins (CDK1, CDK2, CDK4, CDK6, CCNA2, CCNB1, CCNB2, CCNE2, CCNF and CDKN3). These results indicate that the expression of NAA50 was closely related to the cell cycle. Furthermore, CCK-8 assay and Edu assay indicated that knockdown of NAA50 can significantly inhibit the proliferation of lung adenocarcinoma cell lines H1299 and PC9. Therefore, our results manifested that NAA50 may serve as a potential biomarker and therapeutic target for LUAD.

However, although we consolidated information on the role of NAA50 in pan-cancer from multiple databases and verified the cancer promoting effect of NAA50 in lung adenocarcinoma through molecular experiments. The present study still has several limitations. In this study, only the role of NAA50 from different databases in pan-cancer was analyzed by bioinformatics and verified by functional experiments, so further *in vivo* experimental validation and mechanism study are needed. Meanwhile, the exact mechanism supporting the tumor immune effect of NAA50 remains to be clarified.

## Conclusion

In summary, our pan-cancer analysis of NAA50 was conducive to understand the function of NAA50 in tumorigenesis and development from different angles, especially in LUAD. Moreover, NAA50 was bound up with immune cell infiltration in pan-cancer, which would provide a potential immune therapy target for treatment.

## Data Availability

The original contributions presented in the study are included in the article/Supplementary Material, further inquiries can be directed to the corresponding author.

## References

[B1] Aksnes H. Ree R. Arnesen T. (2019). Co-Translational, post-translational, and non-catalytic roles of N-terminal acetyltransferases. Mol. Cell 73 (6), 1097–1114. 10.1016/j.molcel.2019.02.007 30878283 PMC6962057

[B2] Aljohani A. I. Toss M. S. El-Sharawy K. A. Mirza S. Ball G. R. Green A. R. (2022). Upregulation of Cyclin B2 (CCNB2) in breast cancer contributes to the development of lymphovascular invasion. Am. J. Cancer Res. 12 (2), 469–489. 35261781 PMC8899993

[B3] Arnesen T. Van Damme P. Polevoda B. Helsens K. Evjenth R. Colaert N. (2009). Proteomics analyses reveal the evolutionary conservation and divergence of N-terminal acetyltransferases from yeast and humans. Proc. Natl. Acad. Sci. U. S. A. 106 (20), 8157–8162. 10.1073/pnas.0901931106 19420222 PMC2688859

[B4] Chen G. Dong Z. Wu D. Chen Y. (2020). Profiles of immune infiltration in lung adenocarcinoma and their clinical significant: A gene-expression-based retrospective study. J. Cell. Biochem. 121 (11), 4431–4439. 10.1002/jcb.29667 32003059

[B5] Chen S. J. Wang S. C. Chen Y. C. (2021). The immunotherapy for colorectal cancer, lung cancer and pancreatic cancer. Int. J. Mol. Sci. 22 (23), 12836. 10.3390/ijms222312836 34884642 PMC8657810

[B6] Comstock C. E. Augello M. A. Goodwin J. F. de Leeuw R. Schiewer M. J. Ostrander W. F. Jr. (2013). Targeting cell cycle and hormone receptor pathways in cancer. Oncogene 32 (48), 5481–5491. 10.1038/onc.2013.83 23708653 PMC3898261

[B7] Coverley D. Laman H. Laskey R. A. (2002). Distinct roles for cyclins E and A during DNA replication complex assembly and activation. Nat. Cell Biol. 4 (7), 523–528. 10.1038/ncb813 12080347

[B8] Demetriadou C. Pavlou D. Mpekris F. Achilleos C. Stylianopoulos T. Zaravinos A. (2019). NAA40 contributes to colorectal cancer growth by controlling PRMT5 expression. Cell Death Dis. 10 (3), 236. 10.1038/s41419-019-1487-3 30858358 PMC6411749

[B9] Deng S. Magin R. S. Wei X. Pan B. Petersson E. J. Marmorstein R. (2019). Structure and mechanism of acetylation by the N-terminal dual enzyme NatA/naa50 complex. Structure 27 (7), 1057e1054–1070. 10.1016/j.str.2019.04.014 31155310 PMC6610660

[B10] Deng S. McTiernan N. Wei X. Arnesen T. Marmorstein R. (2020). Molecular basis for N-terminal acetylation by human NatE and its modulation by HYPK. Nat. Commun. 11 (1), 818. 10.1038/s41467-020-14584-7 32042062 PMC7010799

[B11] Evjenth R. Hole K. Karlsen O. A. Ziegler M. Arnesen T. Lillehaug J. R. (2009). Human Naa50p (Nat5/San) displays both protein N alpha- and N epsilon-acetyltransferase activity. J. Biol. Chem. 284 (45), 31122–31129. 10.1074/jbc.M109.001347 19744929 PMC2781511

[B12] Feng Z. Chen Y. Cai C. Tan J. Liu P. Chen Y. (2022). Pan-cancer and single-cell analysis reveals CENPL as a cancer prognosis and immune infiltration-related biomarker. Front. Immunol. 13, 916594. 10.3389/fimmu.2022.916594 35844598 PMC9279617

[B13] Gabrilovich D. I. (2017). Myeloid-derived suppressor cells. Cancer Immunol. Res. 5 (1), 3–8. 10.1158/2326-6066.cir-16-0297 28052991 PMC5426480

[B14] Grunwald S. Hopf L. V. M. Bock-Bierbaum T. Lally C. C. M. Spahn C. M. T. Daumke O. (2020). Divergent architecture of the heterotrimeric NatC complex explains N-terminal acetylation of cognate substrates. Nat. Commun. 11 (1), 5506. 10.1038/s41467-020-19321-8 33139728 PMC7608589

[B15] Jia Q. Wang A. Yuan Y. Zhu B. Long H. (2022). Heterogeneity of the tumor immune microenvironment and its clinical relevance. Exp. Hematol. Oncol. 11 (1), 24. 10.1186/s40164-022-00277-y 35461288 PMC9034473

[B16] Jung H. Kim H. S. Kim J. Y. Sun J. M. Ahn J. S. Ahn M. J. (2019). DNA methylation loss promotes immune evasion of tumours with high mutation and copy number load. Nat. Commun. 10 (1), 4278. 10.1038/s41467-019-12159-9 31537801 PMC6753140

[B17] Kalvik T. V. Arnesen T. (2013). Protein N-terminal acetyltransferases in cancer. Oncogene 32 (3), 269–276. 10.1038/onc.2012.82 22391571

[B18] Kim S. M. Ha E. Kim J. Cho C. Shin S. J. Seo J. H. (2020). NAA10 as a new prognostic marker for cancer progression. Int. J. Mol. Sci. 21 (21), 8010. 10.3390/ijms21218010 33126484 PMC7663132

[B19] Knudsen E. S. Kumarasamy V. Nambiar R. Pearson J. D. Vail P. Rosenheck H. (2022). CDK/cyclin dependencies define extreme cancer cell-cycle heterogeneity and collateral vulnerabilities. Cell Rep. 38 (9), 110448. 10.1016/j.celrep.2022.110448 35235778 PMC9022184

[B20] Koufaris C. Kirmizis A. (2021). Identification of NAA40 as a potential prognostic marker for aggressive liver cancer subtypes. Front. Oncol. 11, 691950. 10.3389/fonc.2021.691950 34150665 PMC8208081

[B21] Koufaris C. Kirmizis A. (2020). N-terminal acetyltransferases are cancer-essential genes prevalently upregulated in tumours. Cancers (Basel) 12 (9), 2631. 10.3390/cancers12092631 32942614 PMC7565035

[B22] Li T. Liu T. Zhu W. Xie S. Zhao Z. Feng B. (2021). Targeting MDSC for immune-checkpoint blockade in cancer immunotherapy: Current progress and new prospects. Clin. Med. Insights. Oncol. 15, 11795549211035540. 10.1177/11795549211035540 34408525 PMC8365012

[B23] Liang R. Li X. Li W. Zhu X. Li C. (2021). DNA methylation in lung cancer patients: Opening a "window of life" under precision medicine. Biomed. Pharmacother. 144, 112202. 10.1016/j.biopha.2021.112202 34654591

[B24] Lin X. Xiao M. Chen Z. Ding C. Zhang T. Li Q. (2022). Pancancer analyses reveal genomics and clinical characteristics of the SETDB1 in human tumors. J. Oncol. 2022, 6115878. 10.1155/2022/6115878 35656340 PMC9152430

[B25] Liu W. Liu Y. Hu C. Xu C. Chen J. Chen Y. (2021). Cytotoxic T lymphocyte-associated protein 4 antibody aggrandizes antitumor immune response of oncolytic virus M1 via targeting regulatory T cells. Int. J. Cancer 149 (6), 1369–1384. 10.1002/ijc.33703 34086978

[B26] Liu X. Wu S. Yang Y. Zhao M. Zhu G. Hou Z. (2017). The prognostic landscape of tumor-infiltrating immune cell and immunomodulators in lung cancer. Biomed. Pharmacother. 95, 55–61. 10.1016/j.biopha.2017.08.003 28826097

[B27] Malumbres M. Barbacid M. (2009). Cell cycle, CDKs and cancer: A changing paradigm. Nat. Rev. Cancer 9 (3), 153–166. 10.1038/nrc2602 19238148

[B28] Mughal A. A. Grieg Z. Skjellegrind H. Fayzullin A. Lamkhannat M. Joel M. (2015). Knockdown of NAT12/NAA30 reduces tumorigenic features of glioblastoma-initiating cells. Mol. Cancer 14, 160. 10.1186/s12943-015-0432-z 26292663 PMC4546247

[B29] Najafi M. Majidpoor J. Toolee H. Mortezaee K. (2021). The current knowledge concerning solid cancer and therapy. J. Biochem. Mol. Toxicol. 35 (11), e22900. 10.1002/jbt.22900 34462987

[B30] Nelson A. Lukacs J. D. Johnston B. (2021). The current landscape of NKT cell immunotherapy and the hills ahead. Cancers (Basel) 13 (20), 5174. 10.3390/cancers13205174 34680322 PMC8533824

[B31] Siegel R. L. Miller K. D. Fuchs H. E. Jemal A. (2022). Cancer statistics, 2016. Ca. Cancer J. Clin. 72 (1), 7–30. 10.3322/caac.21332 35020204

[B32] Song C. Guo Z. Yu D. Wang Y. Wang Q. Dong Z. (2020). A prognostic nomogram combining immune-related gene signature and clinical factors predicts survival in patients with lung adenocarcinoma. Front. Oncol. 10, 1300. 10.3389/fonc.2020.01300 32850406 PMC7424034

[B33] Støve S. I. Magin R. S. Foyn H. Haug B. E. Marmorstein R. Arnesen T. (2016). Crystal structure of the golgi-associated human nα-acetyltransferase 60 reveals the molecular determinants for substrate-specific acetylation. Structure 24 (7), 1044–1056. 10.1016/j.str.2016.04.020 27320834 PMC4938767

[B34] Suski J. M. Braun M. Strmiska V. Sicinski P. (2021). Targeting cell-cycle machinery in cancer. Cancer Cell 39 (6), 759–778. 10.1016/j.ccell.2021.03.010 33891890 PMC8206013

[B35] Ugolini F. Pasqualini E. Simi S. Baroni G. Massi D. (2022). Bright-field multiplex Immunohistochemistry assay for tumor microenvironment evaluation in melanoma tissues. Cancers (Basel) 14 (15), 3682. 10.3390/cancers14153682 35954345 PMC9367593

[B36] Witkiewicz A. K. Kumarasamy V. Sanidas I. Knudsen E. S. (2022). Cancer cell cycle dystopia: Heterogeneity, plasticity, and therapy. Trends Cancer 8 (9), 711–725. 10.1016/j.trecan.2022.04.006 35599231 PMC9388619

[B37] Yang H. Li Q. Niu J. Li B. Jiang D. Wan Z. (2016). microRNA-342-5p and miR-608 inhibit colon cancer tumorigenesis by targeting NAA10. Oncotarget 7 (3), 2709–2720. 10.18632/oncotarget.6458 26646451 PMC4823066

